# Mississippi Valley Dental Society

**Published:** 1879-04

**Authors:** 


### Proceedings of Societies.

### MISSISSIPPI VALLEY DENTAL SOCIETY.

*Discussions at the Thirty-fifth Annual Meeting.*

"Chemical Processes that Occur in the Mouth," was the first subject announced for discussion.

Dr. Cassidy was called upon by the chair, but hoped that some one else would start the discussion.

Dr. Taft—The chemical processes that occur in the mouth are various, and there is a variety of substances in the mouth. There is the food, the saliva, the mucous and these mixed, and then the breath with its vapors and odors. These several things are the prominent factors in the chemical changes that occur in the mouth. Changes occur in which all these take a part. Then there is the decomposition or chemical change in the food that remains in the mouth for any considerable length of time, and the character of that change is a very important matter. Then we must take into account that the food is of different kinds—animal and vegetable in various forms. These of course overlap each other, but it is possible to make some analysis of the subject. The most important process, perhaps, is the decomposition of muscle in the mouth. This will ordinarily evolve nitrogen. The starchy material readily undergoes change in the mouth, and this results in the production of grape sugar. That readily undergoes further chemical changes, and results in the formation of acetic acid. Then some of the vegetable substances, as for instance, cabbage, will produce lactic acid; others will produce acetic acid. Other acids are found in the mouth. I suppose acetic acid is frequently formed.

Saliva is not a permanent or fixed material. It is modified by the condition of the blood and the glands at the time of its secretion. If the blood is vitiated the saliva will necessarily vary from its normal condition. Disease of the salivary gland will also produce an abnormal fluid.

Hunger or satiety will cause varying conditions of the saliva. The one producing a distinctly acid character, which the other does not. Its normal condition is either neutral or alkali. It is also modified by age; the saliva of the young differing from that of aged persons.

In early life it is ordinarily able to hold in solution all that is disolved in it, which is not true in later life, precipitation then taking place in a far more marked degree. Then the saliva mixes with the mucous and is subject to thermal changes. It is changed by coming in contact with the atmosphere, independent of thermal conditions. The precipitation on the lingual surface of the inferior front teeth, and the buccal surface of the superior molars, shows that the saliva is not able to hold in solution all the materials contained in it. The mucous also undergoes various changes after it comes into the mouth, in its admixture with the saliva and the food, and its condition is also effected by a vitiated condition of the blood. Another element of importance is the breath. Sometimes very deleterious agents are thrown out by this means. It is impossible by experiments outside the mouth, no matter what the preparation, to accomplish the same results as are going on in the mouth. The conditions are different. Some have the mouth open most of the time when the temperature is but little above the outside temperature; while others are very close mouthed, and then the temperature is about at blood heat. You can, of course, apply nitric acid to the teeth and it will dissolve them, and you can make various compounds that will decompose bone, but none of these processes can be made analogous to the processes that occur in the mouth.

Dr. Cassidy—It seems to me that Dr. Taft has left nothing original for me to say. I agree with him in every thing he has said. The mouth, unlike other cavities, does not possess any special physiological function. The mouth is simply a receptacle and is perfectly helpless in regard to the nature of its own contents. I think caries of the teeth is always produced by chemical reaction in the mouth *in situ*, which takes place principally by fermentation. Every condition necessary for that process is present in the mouth.

Magitot says in his most excellent work, that the acids formed by fermentation are generally analogues to the benzoic or acetic family, and the hemologues. He is right, but he seems to ignore the fact, first introduced by my very much respected friend, Dr. Watt, that the mineral acids, nitric, sulphuric and hydrochloric, are apt to be produced. Owing to the presence of nascent oxygen, nitrogen dioxide is formed, and in the presence of ordinary oxygen, it is readily oxidzed into nitric acid. If sulphur or hydrogen is evolved at the same time, they will also become oxydized; the hydrogen into water, the sulphur into sulphuric acid. There is also at the same time an abundance of free hydrogen. That will take away chlorine and produce hydrochloric acid. Then fermentation of glucose results generally in the formation of acetic acid on the front teeth, and farther back, lactic acid.

Dr. Smith—Does Magitot say that the fermentation of glucose terminates at the alcoholic stage?

Dr. Cassidy—Yes; he seems to ignore the fermentation of acetic acid, although he experiments with it on a tooth outside. Now, I believe that a solvent introduced into the mouth will act upon the teeth by general surface decomposition; greater perhaps in an already existing cavity, and never causes primary decay. We must remember that these bodies produced in the mouth attack the teeth *in situ.*

Dr. Smith—When a patient applies to me with a fine physical organization, teeth of a good structure and good care taken of them, and I see them attacked by caries, it is one of the mysteries to me that I can't comprehend. Is it not possible that they inherit a tendency to acidity of the saliva? Magitot says that the condition of food does not increase the flow from the parotid glands, but that they increase rather from pressure upon them. Dr. Taft spoke of the experiments made out of the mouth. I don't see that they are so different from the changes which take place in the mouth. We have the phosphorus and carbonates, and only the vital element is absent. Of course it may require a stronger condition to overcome the vital element, but otherwise the experiment is the same.

Dr. Morgan—It seems to me that it is very evident to a careful observer that the agents which produce decay result somewhere in defective nutrition, beginning back there. They grow out of our habits of life and mode of feeding, and an evidence of that is, that among savage people, decay of the teeth is very rare. Among the skeletons of the mound builders that I have examined—and they are numerous—I have seen but one single instance of decay, and that was in a very old subject, where the teeth were worn down almost to the alveolar border. Just what the conditions of decay are, are the truths for which we are on the lookout.

Magitot gives another very curious fact which had escaped my observation, and that is the saliva coming from the parotid glands is very different from that produced by the sublinguinal. He asserts that the saliva coming from the sublinguinal glands is ropy and viscid, while that produced from the parotid glands is free from every thing of a mucous character. My observation is not in consonance with his, and if there is a difference I think it is yet to be shown. Another remark has been made which my observation does not bear out; and that is, that the saliva of aged persons has less power to hold in solution whatever is made part of it, than is the saliva of younger persons. If that were so, I think it would be proven by the increased deposit of salivary calculus upon them; and that I think is not so. In fact, there is a much larger flow of saliva in the young, and especially a much larger flow of mucous. Another position of Dr. Taft was, that where there was a vitiated condition of the blood the saliva would be abnormal. I don't so understand it. There might be a case where there would be a superabudance of the material that produces saliva, and in that case we would have an abnormal flow, but still the fluid remains in its normal condition.

Dr. Leslie thought the teeth the most remarkable organs of the human system from the fact that they were produced twice. He thought the effect of acids upon the teeth a subject destined to be of far greater interest to dentists than it has been heretofore.

Dr. J. Taft—Dr. Smith expressed surprise at many cases in which he found decay of teeth where they were well formed and cared for, about which no accumulation is permitted. Two or three things have to be taken into account. In such cases there can not be much decay. Decay does occur sometimes, but let us not be mistaken because it looks well, and presume that therefore it is perfect in its structural make-up. Take any tooth however perfect it may seem on the surface, and make a section from it, and examine it thoroughly and there will be more or less of imperfect tracks in it, that will apply both to the enamel and dentine. I wish to emphasize the remark of Dr. Cassidy, that all decay is from the nascent form of acid. Now, as to the statement of Dr. Morgan that the fluids of the mouth are not modified by a vitiated condition of the blood.

Dr. Morgan—I said that it may not necessarily be in all cases.

Dr. Taft—If a vitiated condition of the blood does not affect the saliva that is drawn from it, how is it that the function of various organs become excited or depressed by systemic treatment? Is it not through the medium of the blood?

Does not the presence of certain substances in the blood excite the liver to increased action? And under that action is there no variation in the product?

Does not certain substances in the blood excite the action of the kidneys and with the same results?

Now I simply stated that when the blood is vitiated, the secretions of the mouth are changed from the normal state. The consideration of this subject leads us to this point; we must understand the general principles pertaining to, and character of the blood, the source of these secretions, the character of the secreting apparatus and its function, so far as possible, together with the responses which it makes to the various influences that may be made to bear upon it.

The hour of adjournment having arrived, the society adjourned to meet at half past one p. m.

TO BE CONTINUED.

